# Acute Vitamin D Intoxication Possibly Due to Faulty Production of a Multivitamin Preparation

**DOI:** 10.4274/Jcrpe.89

**Published:** 2013-05-30

**Authors:** Ahmet Anık, Gönül Çatlı, Ayhan Abacı, Ceyhun Dizdarer, Ece Böber

**Affiliations:** 1 Dokuz Eylül University Faculty of Medicine, Department of Pediatric Endocrinology, İzmir, Turkey; 2 Dr. Behçet Uz Children’s Research and Training Hospital, Department of Pediatric Endocrinology, İzmir, Turkey

**Keywords:** Vitamin D intoxication, hypercalcemia, multivitamin preparation

## Abstract

Vitamin D intoxication usually occurs as a result of inappropriate use of vitamin D preparations and can lead to life-threatening hypercalcemia. It is also known that there are a number of physicians who prescribe vitamin D supplements for various clinical conditions, such as poor appetite and failure to thrive. While inappropriate use of vitamin D supplements may lead to vitamin D intoxication, there are no reports of cases of vitamin D toxicity due to manufacturing errors of vitamin D preparations. Here, we present cases of hypervitaminosis D which developed following the use of a standard dose of a multivitamin preparation. All three cases presented with hypercalcemia symptoms and had characteristic laboratory findings such as hypercalcemia, hypercalciuria, low levels of parathyroid hormone. The very high serum 25(OH) vitamin D levels in these patients indicated vitamin D excess. The vitamin D level of the prescribed multivitamin preparation in the market was studied and was found to contain a very low level of vitamin D (10 IU/5 mL). Although the stated vitamin D content of the preparations ingested by these patients was not high, unproven but possible manufacturing errors were considered to be a possible cause of the hypervitaminosis D diagnosed in these three patients.

**Conflict of interest:**None declared.

## INTRODUCTION

Vitamin D intoxication usually occurs as a result of improper use of pharmaceutical preparations of vitamin D and can lead to life-threatening hypercalcemia (1). Irrational prescribing of this vitamin for unproven rickets is an important health care problem in Turkey and may lead to unintentional poisoning (2). Vitamin D poisoning has been associated with excessively fortified milk, over-the-counter (OTC) supplements, contamination of cooking nut oil, and table sugar (1). Furthermore, in two adult cases, vitamin D intoxication with severe hypercalcemia due to manufacturing error of dietary supplements has been reported recently (3). To the best of our knowledge, there are no reported cases of vitamin D intoxication in infants or children due to manufacturing errors of supplements. We present three cases of vitamin D toxicity due to suspicious manufacturing errors of prescribed multivitamin supplements.

## CASE 1

A 19-month-old girl with no previous health problems presented with abdominal pain, vomiting, and poor appetite. The medical history revealed that she had been given a multivitamin preparation once daily (containing 200 IU/day vitamin D) for a month, prescribed by her physician for poor appetite. The patient’s body weight was 7.1 kg (-4.7 SDS), height 71 cm (-2.9 SDS), and head circumference 44 cm (-1.9 SDS). On physical examination, systemic signs were normal. Laboratory parameters were Ca: 19.4 mg/dL (N=8.4-10.2), P: 2.9 mg/dL (N=2.7-4.5), creatinine: 0.3 mg/dL (N=0.5-1.3), PTH: 3.3 pg/mL (N=15-65), 25(OH) vitamin D: 760 ng/mL (N=25-80), and urinary Ca/creatinine ratio: 1.4 mg/mg (N<0.21).The patient was treated with 3000 mL/m2/day intravenous (IV) hydration and 2 mg/kg/day furosemide, with dietary restrictions of calcium. Renal ultrasonography showed an image consistent with bilateral nephrocalcinosis. Serum Ca was 16.5 mg/dL at 24 hours, and prednisolone 1 mg/kg/day was also added to the treatment regimen. On day 3, the patient received pamidronate IV 1 mg/kg over 6 hours due to her persistent hypercalcemia. On day 4, serum Ca level was 12.8 mg/dL, and the patient was discharged on day 7 with a serum Ca level of 10.5 mg/dL. The patient did not present for her follow-up appointment after discharge.

## CASE 2

A 25-month-old boy with no previous health problem was admitted with loss of appetite, failure to gain weight, and vomiting in the past two weeks. Patient history revealed regular multivitamin consumption of 5 mL per day, which is known to contain 200 IU vitamin D, in the past two weeks. The preparation was prescribed by her physician as a dietary supplement. Physical examination revealed a body weight of 12.7 kg (+0.18 SDS), height of 88 cm (+0.23 SDS), and head circumference of 50 cm (+1.59 SDS). Systemic findings were normal. Laboratory parameters were Ca: 19.3 mg/dL, P: 7.7 mg/dL, creatinine: 0.5 mg/dL, PTH: <3 pg/mL, 25(OH) vitamin D >160 ng/mL, and urinary Ca/creatinine ratio: 1.03 mg/mg. The patient was treated with 3000 mL/m2/day IV hydration and 1 mg/kg/day furosemide with dietary restrictions for two days. Renal ultrasonography showed normal findings and no nephrocalcinosis. However, serum Ca level was 16.5 mg/dL at 24 hours. Prednisolone 1 mg/kg/day was also added. On day 4, serum Ca level was 12.8 mg/dL. The patient was discharged on day 7 with a serum Ca level of 10.5 mg/dL. Serum Ca and vitamin levels were within the normal range at the follow-up visit.

## CASE 3

A one-year-old boy was admitted with complaints of irritation, constipation, loss of appetite, and vomiting for 20 days. Patient history revealed regular consumption of a multivitamin preparation, 5 mL per day known to contain 200 IU vitamin D, in the past ten days, prescribed as a dietary supplement. Body weight was 10 kg (-0.14 SDS), and height was 80 cm (+1.65 SDS). Systemic signs were normal. Laboratory parameters were Ca: 13.7 mg/dL, P: 4.1 mg/dL, creatinine: 0.46 mg/dL, PTH <3 pg/mL, 25(OH) vitamin D >160 ng/mL, and urinary Ca/creatinine ratio: 1.2 mg/mg. The patient received therapy with 3000 mL/m2/day IV hydration and 1 mg/kg/day furosemide with dietary restrictions for two days. Renal ultrasonography showed an image consistent with bilateral nephrocalcinosis. Serum Ca level was 14.1 mg/dL at 24 hours. Prednisolone 1 mg/kg/day was also added. Patient was discharged on day 3 with a serum Ca level of 11 mg/dL. Serum Ca and vitamin levels were within the normal range at the follow-up visit.The biochemical findings in the 3 patients are given in Table 1.

## DISCUSSION

Vitamin D increases calcium absorption from the gastrointestinal system and enhances bone resorption. Vitamin D intoxication occurs secondarily from excessive uptake of vitamin D. The upper limit of vitamin D uptake for long-term use is 1000 IU/day for patients less than 1 year old and 2000 IU/day for older children (1). In many reported cases, intoxication is associated with accidental excessive uptake of prescribed vitamin D or consumption of OTC supplements containing vitamin D in high doses (4).

In developed countries, vitamin D intoxication due to manufacturing errors of dairy products during the enrichment process of vitamin D to reduce nutritional rickets has been reported (5). In our country, hypervitaminosis D is usually associated with unnecessary prescription of vitamin D at improper doses and duration in patients who present with loss of appetite, developmental and growth retardation, wide anterior fontanelle, and retardation in eruption of the teeth (2). All three patients presented in this report had been prescribed by physicians a multivitamin preparation containing vitamin D without any investigation for a specific diagnosis.

Symptoms of vitamin D intoxication including loss of appetite, vomiting, constipation, growth retardation, polyuria, dehydration, and fever are secondary to hypercalcemia. Characteristic laboratory findings are hypercalciuria and nephrocalcinosis due to hypercalcemia and parathormone suppression with an elevated level of 25(OH) vitamin D (>150 ng/mL) (1). In our study, all cases presented with loss of appetite and vomiting, indicating typical vitamin D intoxication as assessed by laboratory findings. In two of these patients, renal ultrasonography showed an image consistent with bilateral nephrocalcinosis. Three months after discharge, the level of serum vitamin D and calcium were lowered to normal limits in two cases. The data of the other case were missing due to nonattendance of the patient. All three patients had been prescribed multivitamin preparations containing vitamin D (200 IU) by different physicians. Also, all 3 patients had received the multivitamin preparation in the same dosage (vitamin D 200 IU/day) without any additional supplement containing vitamin D. Thus, the observation that, within the same time period (between October and December 2011), the same preparation with identical recommended doses given to these patients caused vitamin D intoxication led us to think that the amount of vitamin D in this pharmaceutical product might be higher than the specified amount. Since the preparations ingested by the patients were not available, the vitamin D level of the multivitamin preparation in the market was assessed using the High Performance Liquid Chromatography method, following an extraction process. It was established that the ingredient vitamin D present in the product was 25(OH) vitamin D and that the product contained a low level of vitamin D (10 IU/5 mL). Although the vitamin D content of the preparations investigated was not high, preparations used by patients and those examined at the laboratory were not the same. It may be hypothesized that the preparations ingested by the patients might have had higher levels of vitamin D due to manufacturing errors at that time interval, which might have led to intoxication. Recently, Araki et al (3) reported two adult cases of vitamin D intoxication with dietary supplements caused by significant manufacturing and labeling errors that led to 1000 fold higher levels in vitamin D content.

As the hypercalcemic response to vitamin D may be variable, the dose used in the treatment and response to treatment may widely differ (6). Differences in treatment response may be associated with absorption of vitamin D, serum vitamin D-binding protein level, and storage capacity of vitamin D (6). Vanstone et al (7) reported mild hypercalcemia and moderate elevation of 25(OH) vitamin D in three infants with rickets who received vitamin D in doses of 1400 IU/day, 2000 IU/day, and 2000 IU/day. These authors recommend that the vitamin D doses used in treatment of childhood rickets should be reassessed. However, the authors did not believe that individual sensitivity was associated with intoxication, since all three cases showed moderate hypercalcemia (10.4-10.9 mg/dL), moderate elevation of 25(OH) vitamin D (79-102 ng/mL), and no signs of vitamin D intoxication. Thus, the very high level of serum vitamin D levels and significant toxicity observed in our cases cannot be explained with individual response to physiological doses of vitamin D.

In the treatment of vitamin D intoxication, exogenous vitamin D intake is discontinued, and a regimen consisting of intravenous fluid therapy, loop diuretics, glucocorticoids, and a calcium-restricted diet is applied. In addition, calcitonin and bisphosphonates (pamidronate) may be used, particularly in severe cases (1,2,8). Hemodialysis may be effective in reducing the serum calcium level rapidly in hypercalcemic patients who are refractory to the treatment (2). In our patients, the calcium levels could not be reduced sufficiently with intravenous hydration, loop diuretics, and a calcium-restricted diet. Prednisolone was also added. Two cases became normocalcemic with glucocorticosteroid treatment, while bisphosphonate was used in one case.

In Turkey, since 2005, according to the recommendations of the Turkish Ministry of Health, vitamin D supplements (400 IU/day) are regularly prescribed to infants, free of charge in order to reduce the prevalence of nutritional rickets (9). In addition, advertisements on multivitamin preparations containing vitamin D in the visual and printed media are misleading and induce individuals to believe that these preparations are not drugs, but “nutritional supplements” thus increasing the demand for these products. We believe that these preparations should not be used, unless they are evidence-based, since off-label use may lead to intoxication.

In conclusion, the findings in these three patients led us to underline that apart from regularly prescribed daily vitamin D prophylactic use, a vitamin D supplement should be prescribed only to patients diagnosed to have rickets, based on their clinical and laboratory findings. Secondly, we would like to stress that multivitamin preparations should not be used for vitamin D treatment and that patients unnecessarily treated with vitamin D need to be evaluated for findings of hypervitaminosis.

## Figures and Tables

**Table 1:**
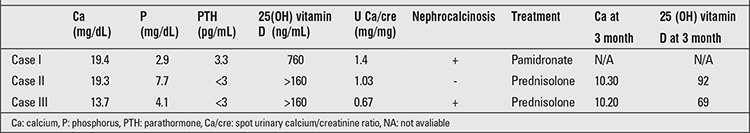
Biochemical characteristics of the patients and renal ultrasonography findings at admission and 3 months after discharge

## References

[1] Greenbaum LA. Hypervitaminosis D, Kliegman RM, Stanton BF, Geme JW, Schor NF, Behrman RE (2011). Nelson Textbook of Pediatrics. Nelson Textbook of Pediatrics.

[2] Ozkan B, Hatun S, Bereket A (2012). Vitamin D intoxication. Turk J Pediatr.

[3] Araki T, Holick MF, Alfonso BD, Charlap E, Romero CM, Rizk D, Newman LG (2011). Vitamin D intoxication with severe hypercalcemia due to manufacturing and labeling errors of two dietary supplements made in the United States. J Clin Endocrinol Metab.

[4] Sezer RG, Guran T, Paketci C, Seren LP, Bozaykut A, Bereket A (2012). Comparison of oral alendronate versus prednisolone in treatment of infants with vitamin D intoxication. Acta Paediatr.

[5] Blank S, Scanlon KS, Sinks TH, Lett S, Falk H (1995). An outbreak of hypervitaminosis D associated with the overfortification of milk from a home-delivery dairy. Am J Public Health.

[6] Plotkin H, Lifshitz F, Lifshitz F (2009). Rickets and osteoporosis. Pediatric Endocrinology.

[7] Vanstone MB, Oberfield SE, Shader L, Ardeshirpour L, Carpenter TO (2012). Hypercalcemia in children receiving pharmacologic doses of vitamin D. Pediatrics.

[8] Barrueto F Jr, Wang-Flores HH, Howland MA, Hoffman RS, Nelson LS (2005). Acute vitamin D intoxication in a child. Pediatrics.

[9] Hatun S, Ozkan B, Bereket A (2011). Vitamin D deficiency and prevention Turkish experience.. Acta Pædiatr.

